# Quantitative Profiling of DNA Damage and Apoptotic Pathways in UV Damaged Cells Using PTMScan Direct

**DOI:** 10.3390/ijms14010286

**Published:** 2012-12-21

**Authors:** Matthew P. Stokes, Jeffrey C. Silva, Xiaoying Jia, Kimberly A. Lee, Roberto D. Polakiewicz, Michael J. Comb

**Affiliations:** Cell Signaling Technology, 3 Trask Lane, Danvers, MA 01923, USA; E-Mails: jsilva@cellsignal.com (J.C.S.); xjia@cellsignal.com (X.J.); kimberly.lee@cellsignal.com (K.A.L.); rpolakiewicz@cellsignal.com (R.D.P.); mcomb@cellsignal.com (M.J.C.)

**Keywords:** proteomics, liquid chromatography tandem mass spectrometry, DNA damage response, apoptosis, post-translational modification, PTMScan direct

## Abstract

Traditional methods for analysis of peptides using liquid chromatography and tandem mass spectrometry (LC-MS/MS) lack the specificity to comprehensively monitor specific biological processes due to the inherent duty cycle limitations of the MS instrument and the stochastic nature of the analytical platform. PTMScan Direct is a novel, antibody-based method that allows quantitative LC-MS/MS profiling of specific peptides from proteins that reside in the same signaling pathway. New PTMScan Direct reagents have been produced that target peptides from proteins involved in DNA Damage/Cell Cycle and Apoptosis/Autophagy pathways. Together, the reagents provide access to 438 sites on 237 proteins in these signaling cascades. These reagents have been used to profile the response to UV damage of DNA in human cell lines. UV damage was shown to activate canonical DNA damage response pathways through ATM/ATR-dependent signaling, stress response pathways and induce the initiation of apoptosis, as assessed by an increase in the abundance of peptides corresponding to cleaved, activated caspases. These data demonstrate the utility of PTMScan Direct as a multiplexed assay for profiling specific cellular responses to various stimuli, such as UV damage of DNA.

## 1. Introduction

Cells have evolved complex mechanisms to halt cell cycle progression in response to genomic insult, collectively termed the DNA damage checkpoint [1–3]. These checkpoint controls allow time for repair or bypass of the lesioned DNA, or, if the damage is too severe, to activate the apoptotic program [4–8]. The Phosphatidylinositol 3-kinase-like kinases ATM and ATR are central upstream regulators of the DNA damage checkpoint, phosphorylating consensus SQ or TQ motifs on downstream effectors, such as the kinases Chk1 and Chk2 and the transcriptional regulator p53 [1,9–27]. Chk1 and Chk2 kinases in turn phosphorylate substrates, including p53 and the Cdc25 phosphatases, which halt cell cycle progression through inhibition of cyclin dependent kinase activity [14,21,28–33].

In addition to the core DNA damage checkpoint pathway, hundreds of other proteins have been identified that participate in a more generalized DNA damage response (DDR) [3,34–36]. Proteomic analyses of DNA damage signaling using liquid chromatography-tandem mass spectrometry (LC-MS/MS) have identified hundreds of candidate ATM/ATR substrates more highly phosphorylated in response to damage [37–40]. These studies, along with years of more focused biochemical analysis, emphasize the far-reaching effects of the DDR in affecting protein post-translational modification across many signaling pathways controlling diverse cellular functions.

In addition to studies of the DNA damage response, antibody-based proteomic methods have also been employed to study signaling areas, such as cancer, neurodegenerative disease, growth and development and infectious disease [37,39–52]. Other LC-MS-based proteomic methods of PTM analysis have employed sample fractionation and whole proteome analysis or global phosphopeptide enrichment methods, such as immobilized metal affinity chromatography (IMAC) with chelated iron or titanium dioxide beads [38,53–66]. Such fractionation and enrichment strategies, combined with the sensitivity and speed of current LC-MS/MS systems, allow identification of hundreds to thousands of post-translationally modified peptides in a single LC-MS/MS run. These methods suffer, however, from the fact that the peptides identified and quantified are randomly sampled by the instrument and tend to focus on the more abundant peptides present in a sample, while critical signaling may be mediated by proteins expressed at exquisitely low levels. The results from these proteomic studies can therefore lack the specificity necessary to probe signaling networks in enough detail to sufficiently understand the mechanism of action or cellular response to a particular perturbation.

A novel method for identification and quantification of post-translationally modified peptides, termed PTMScan Direct, was recently published [67]. This method enables focused study of peptides from proteins in the same signaling pathway or from the same protein type. Four PTMScan Direct reagents were previously designed that probed critical signaling nodes of many different pathways (the Multipathway Reagent), phosphorylation sites on kinases (the Ser/Thr Kinases Reagent and Tyr Kinases Reagent) and Phosphatidylinositol 3-kinase signaling networks (the Akt/PI3K Pathway Reagent). Each reagent allows quantification of hundreds of post-translationally modified peptides from proteins that reside in the desired signaling space in a single LC-MS/MS run. PTMScan Direct can be used on cell lines, tissues, xenografts or primary cells. The method has been validated for use in humans and mice, but could be extended to include any species with an available proteomic database (for peptide identification). This method therefore combines the specificity of traditional biochemical analysis with the speed, sensitivity and high number of endpoints inherent to LC-MS/MS analysis.

Two new PTMScan Direct Reagents have been formulated that target DNA damage and cell cycle pathways, as well as apoptotic and autophagolytic pathways (PTMScan Direct: DNA Damage/Cell Cycle and Apoptosis/Autophagy Reagents, respectively). Each PTMScan Direct reagent allows in-depth analysis of its targeted pathways by focusing the MS sampling on the immunoaffinity-enriched target peptides, eliminating the random sampling that can occur with traditional global enrichment strategies. Reagents were rigorously validated using both human samples, as well as mouse tissues, with only peptides that pass strict criteria (MS/MS score filtering; intensity filters; enrichment *versus* control filters; presence of a sequence targeted by the reagent, *etc.*) accepted as validated targets. The DNA Damage/Cell Cycle Reagent allows identification of 263 sites on 137 proteins, while the Apoptosis/Autophagy Reagent targets 175 sites of phosphorylation and caspase cleavage activation sites on 100 proteins.

These unique reagents have been used to study activation of the DNA damage checkpoint and induction of apoptosis in HeLa cells damaged with UV light. In response to UV damage of DNA, peptides from markers of checkpoint signaling (ATM, Chk1, Chk2), cell cycle arrest (p21), stress response (p38 MAPK, JNK) and apoptosis (caspase cleavage, cytochrome c) all changed in abundance in the PTMScan Direct studies. Changes to selected signaling nodes were confirmed by western blotting, highlighting the accuracy of the method. These reagents therefore provide detailed, reproducible analysis of hundreds of peptides from proteins involved in the response to DNA damage and damage-induced cell death in a single LC-MS/MS run and are powerful tools for LC-MS/MS-based proteomic analysis.

## 2. Results

### 2.1. PTMScan Direct: DNA Damage/Cell Cycle and Apoptosis/Autophagy Reagents

Two new PTMScan Direct reagents have been developed to study the DNA damage response and cell cycle progression, and apoptosis and autophagy pathways. Reagents were prepared with a focus on proteins and sites in curated pathways, such as those found at www.cellsignal.com (Cell Signaling Technology, Danvers, MA, USA. Both reagents were validated using a strategy outlined previously [67]. Briefly, reagents were used to immunoaffinity purify (IAP) peptides from a mixture of human cancer cell lines treated with pervanadate to artificially increase phosphopeptide levels (“CST Cell Line” in Tables S1–S4), as well as mouse liver, brain and embryo samples [67] (data not shown). Jurkat cells treated with pervanadate or etoposide were also run as validation samples (see Tables S1 and S2). To be considered validated, the peptides identified had to pass a number of filters, including score filtering of the MS/MS assignment, a minimum MS1 signal intensity filter (minimum 20,000 for MS1 peak height from XCalibur processing (v2.0.7; Thermo Fisher Scientific, Barrington, IL, USA)) and a higher abundance of the peptide in the PTMScan Direct Reagent IAP than in a control IAP using empty protein G beads. Candidate peptides were then limited to those originally targeted by the reagent or homologous to a target and peptides from proteins involved in the signaling area of interest. Complete lists of peptides validated using this strategy with accompanying MS/MS scoring metrics are available in Tables S1 and S2.

**Figure 1:**
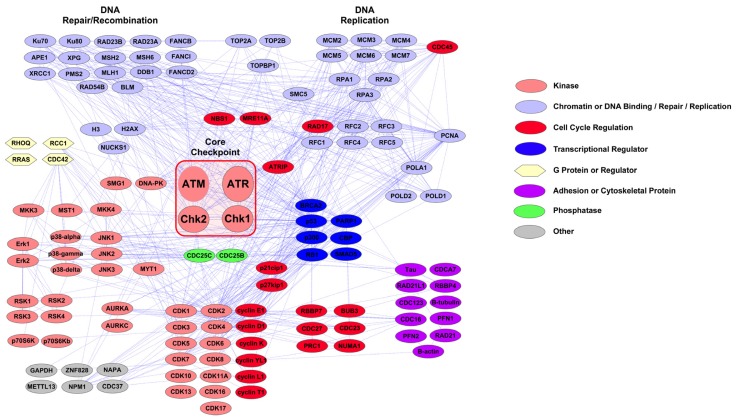
PTMScan Direct: DNA Damage/Cell Cycle Reagent Interaction Map. Interactions were derived from the STRING database using experimental, database and text-mining lines of evidence and high confidence scores (score > 0.700). Interactions were imported into Cytoscape 2.8.1 for map generation. Protein color and shape denote protein class, as detailed in the legend to the right. Core checkpoint proteins ATM, ATR, Chk1 and Chk2 are highlighted with a red box in the center of the map.

Proteins for which peptides can be identified using the DNA Damage/Cell Cycle Reagent are shown in Figure 1. Interactions are from the STRING database, and pathway maps were created in Cytoscape 2.8.1 [68,69]. The interaction map in Figure 1 is organized by protein function, with the core checkpoint proteins ATM, ATR, Chk1 and Chk2 in the center of the map highlighted by a red box. The reagent goes well beyond identification of canonical checkpoint proteins to cover other DNA damage response proteins, including those involved in stress response, DNA repair, DNA replication, transcriptional regulation, adhesion and cytoskeletal organization, and cell cycle control. Overall, peptides representing 263 sites on 137 proteins were validated for the DNA Damage/Cell Cycle Reagent. A full list of proteins and sites identified using the reagent is available in Table S1.

**Figure 2:**
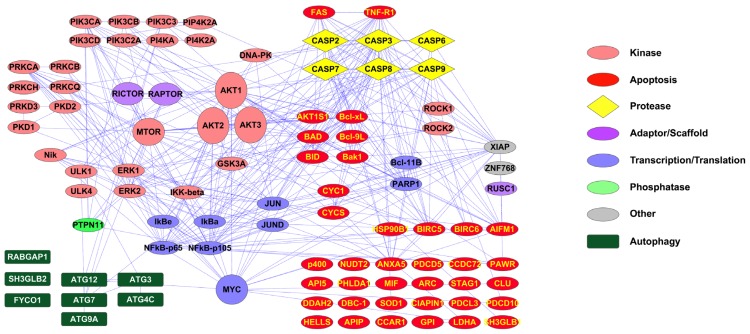
PTMScan Direct: Apoptosis/Autophagy Reagent Interaction Map. Interactions were derived from the STRING database using experimental, database and text-mining lines of evidence and high confidence scores (score > 0.700). Interactions were imported into Cytoscape 2.8.1 for map generation. Protein color and shape denote protein class, as detailed in the legend to the right.

The interaction map in Figure 2 shows proteins for which peptides can be identified using the PTMScan Direct: Apoptosis/Autophagy Reagent. As in Figure 1, interactions are from the STRING database imported into Cytoscape 2.8.1. The reagent targets peptides from a number of autophagy-specific factors, such as ATG proteins, kinases, including Akt, Erk, PKC and IKK, transcription/translation proteins, such as IkBa, NFkB, myc and Jun, and a large number of apoptotic proteins, including caspases, BAD, Bcl-xL, cytochrome c and FAS and TNF receptors. The caspase peptides targeted include sites of caspase cleavage and activation, such as Asp175 of caspase 3, Asp179 of caspase 6, Asp198 of caspase 7, Asp374 of caspase 8 and Asp315 of caspase 9. All peptides validated as targets of the reagent are shown in Table S2.

### 2.2. PTMScan Direct Profiling of UV Damage in HeLa Cells

**Figure 3:**
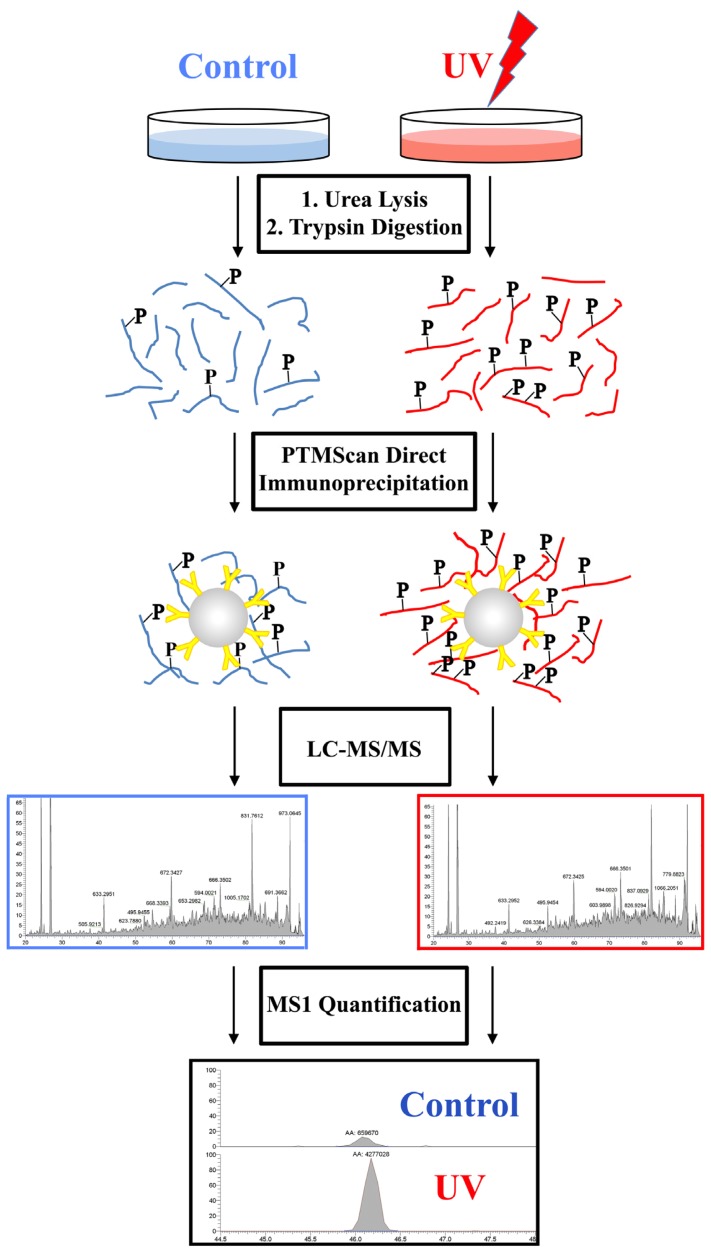
HeLa –/+ UV PTMScan Direct Experimental Design. HeLa cells were untreated or treated with 50 mJ/cm^2^ UV light and harvested at 2 h post treatment. Cells were processed according to previous protocols [67] and protein lysates were digested with trypsin. Immunoaffinity purification of peptides was performed with either the DNA Damage/Cell Cycle Reagent or the Apoptosis/Autophagy Reagent. Eluted peptides were run in LC-MS/MS, peptides were identified using Sorcerer 2 [70], and label-free quantification was performed using peptide chromatographic peak heights or areas in the MS1 channel.

The two reagents were used to assess activation of the DNA damage response and onset of apoptosis in HeLa cells treated with 50 mJ/cm^2^ UV light and harvested 2 h post-treatment. These treatment conditions are effective in causing significant DNA damage and activation of checkpoint signaling [40]. Cells were harvested, digested and immunoprecipitated with each of the PTMScan Direct reagents in parallel as outlined in Figure 3. Replicate immunoaffinity purifications were performed with each reagent, and each sample was injected on the LC-MS/MS in duplicate. The complete data tables including scoring of the MS/MS assignments and label-free quantification are available as Table S3 for the DNA Damage/Cell Cycle Reagent and Table S4 for the Apoptosis/Autophagy Reagent.

**Figure 4:**
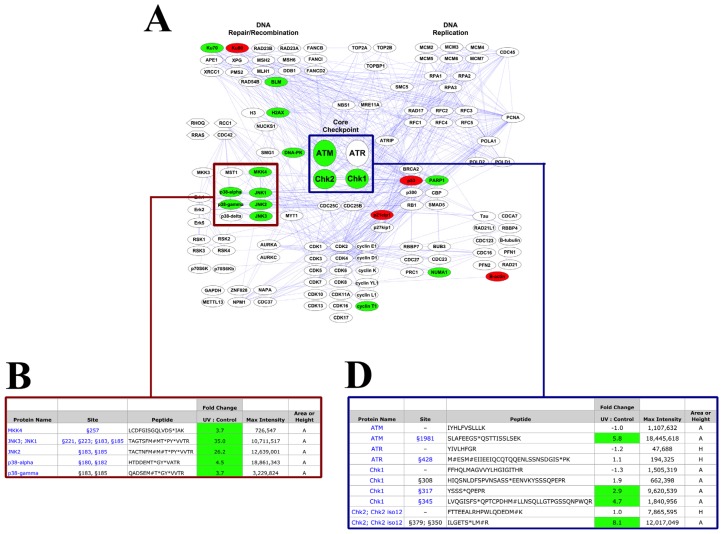

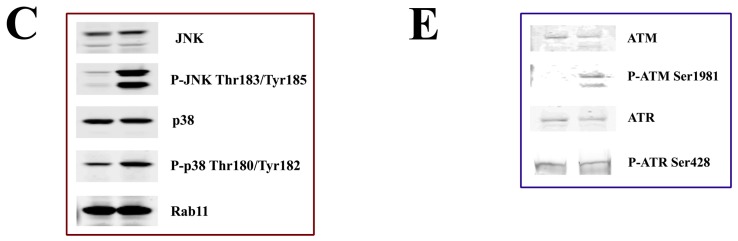
DNA Damage/Cell Cycle Reagent HeLa –/+ UV Results. (**A**) Quantitative data from replicate runs was mapped onto the pathway diagram from Figure 1. Green proteins denote peptides that increased at least 2.5-fold with UV damage, red proteins denote decreases of at least 2.5-fold. The red box highlights stress-responsive MAP kinase proteins, and the blue box highlights core DNA damage checkpoint proteins; (**B**) Selected peptides derived from p38 MAPK and JNK are shown in detail with modification/cleavage site number, peptide sequence, fold change (UV: Control), maximum intensity and whether the measurement was height (H) or area (A). Green cells represent peptides that increased with UV damage at least 2.5-fold. Total and phospho-specific antibodies are available for protein names and sites shown in blue text, respectively; (**C**) Western blotting of the same samples used for the PTMScan Direct analysis with total and phospho-specific p38 and JNK antibodies (Cell Signaling Technology). Rab11 is included as a total protein control; (**D**) Selected peptides derived from the checkpoint proteins ATM, ATR, Chk1 and Chk2 are shown as in part B. “–” in the site column indicates an unmodified peptide; (**E**) Western blotting of the same samples used for the PTMScan Direct analysis with total and phospho-specific ATM and ATR antibodies (Cell Signaling Technology).

The interaction map for the DNA Damage/Cell Cycle Reagent shown in Figure 1 is replicated in Figure 4A, color-coded by changes in peptide abundance in response to UV damage of HeLa cells. Proteins from which peptides were identified that increased in abundance at least 2.5-fold with UV damage are shown in green, while red signifies decreases in abundance of at least 2.5-fold. Selected peptides from proteins highlighted in Figure 4A with a red box are shown in greater detail in Figure 4B. The highlighted peptides are derived from the stress-responsive MAP kinases p38a, p38g, JNK1, JNK2, JNK3 and the upstream MAP kinase-kinase MKK4. Each of these phosphopeptides increased in abundance with UV damage, from 3.7- to 35.0-fold. Western blots in Figure 4C confirmed that, while there was no change in total levels of p38 or JNK, their phosphorylation at critical activation loop residues [71–75] did in fact increase, consistent with the PTMScan Direct data.

The blue box in Figure 4A highlights the core checkpoint proteins ATM, ATR, Chk1 and Chk2. Unmodified and phosphorylated peptides from these proteins are shown in Figure 4D (unmodified peptides are those with “–” in the Site column of Figure 4D). In each case, the unmodified peptides did not change in abundance with UV damage (1.3- to 1.0-fold change), while the phosphopeptides (ATM Ser1981, Chk1 Ser317 and Ser345, and Chk2 Ser379) by contrast all increased in abundance with UV damage (2.9- to 8.1-fold). The ATR peptide phosphorylated at Ser428 did not show an increase with UV damage in these cells (1.1-fold change). Western blots in Figure 4E are again consistent with the PTMScan Direct data, showing no change in protein level as assessed by the total ATM and ATR antibodies, an increase in signal with the ATM S1981 phospho-specific antibody and no change in signal with the ATR S428 phospho-specific antibody.

Other checkpoint proteins were also affected by damage, including the ATM/ATR family member DNA-PK, from which a Ser3205-phosphorylated peptide was identified that increased 6.2-fold with UV treatment. The DNA damage-associated histone, histone H2AX [76,77], was identified singly phosphorylated at Thr137 and dually phosphorylated at Thr137/Ser140, and both the singly and doubly-phosphorylated peptides increased with damage (4.9-fold increase and 5.1-fold increase, respectively). p21cip1 is a tumor suppressor protein that blocks cell cycle progression through inhibition of cyclin-dependent kinase (Cdk) and PCNA activity [78–81]. Unmodified peptides from the p21cip1 protein, as well as a Ser130 phosphopeptide, were identified and all decreased with UV treatment, from -4.8- to -10.8-fold.

**Figure 5:**
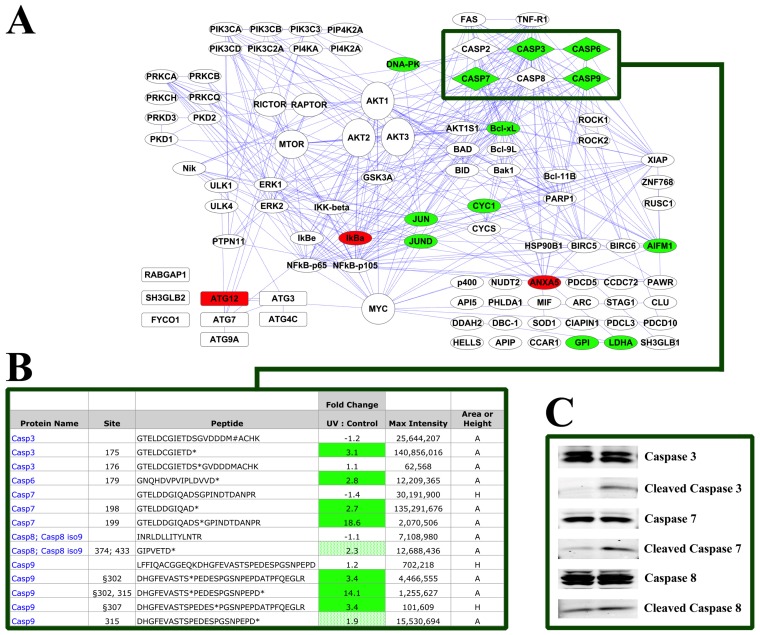
Apoptosis/Autophagy Reagent HeLa −/+ UV Results. (**A**) Quantitative data from replicate runs was mapped onto the pathway diagram from Figure 2. Green proteins denote peptides that increased at least 2.5-fold with UV damage, and red proteins denote decreases of at least 2.5-fold. The green box highlights caspase proteins; (**B**) Selected peptides derived from caspases are shown in detail with modification/cleavage site number, peptide sequence, fold change (UV: Control), maximum intensity and whether the measurement was height (H) or area (A). Green cells represent peptides that increased with UV damage at least 2.5-fold. Light green cells indicate increases in cleaved caspase peptides less than 2.5-fold. Total antibodies are available for protein names shown in blue text; (**C**) Western blotting of the same samples used for the PTMScan Direct analysis with caspase 3, 7 and 8 antibodies (Cell Signaling Technology).

The quantitative data collected using the Apoptosis/Autophagy Reagent is shown mapped onto the pathway diagram for the reagent in Figure 5A. As in Figure 4A, proteins with peptides that increased in abundance over 2.5-fold with UV treatment are shown in green, while decreases of at least 2.5-fold are shown in red. Selected caspase peptides highlighted in Figure 5A with a green box are shown in detail in Figure 5B. Peptides containing the cleavage and activation sites increase with damage for caspase 3 (3.1-fold), caspase 6 (2.8-fold) and caspase 7 (2.7-fold). The peptide derived from a cleaved form of caspase 8 increased slightly (2.3-fold), as did the cleaved peptide for caspase 9 (1.9-fold). None of the unmodified/uncleaved caspase peptides changed to this level with damage (max fold change = −1.4). Phosphorylated caspase peptides were also identified, and some increased with UV treatment including caspase 7 Ser199 (18.6-fold) and caspase 9 Ser302 and Ser307 (3.4-fold change for both). A phosphorylated caspase 9 peptide cleaved at Asp315 was also identified and increased significantly with UV damage (14.1-fold). Unlike the other phosphorylated caspase peptides, the S176-phosphorylated caspase 3 peptide did not change with damage (1.1-fold change). Western blots using caspase antibodies (Figure 5C) confirmed the PTMScan Direct results, with no change in full-length caspase 3, 7 or 8 protein levels, but a modest increase in the cleaved forms of caspase 3 and 7, and a slight increase in the cleaved form of caspase 8.

Figure 5A also highlights changes to other proteins beyond the caspases. c-Jun and JunD phosphopeptides both increased with UV treatment. Ser58, Thr62, Ser63 and Ser73-containing peptides for c-Jun increased in abundance 3.7- to 22.6-fold. A JunD peptide phosphorylated at Ser100 increased 9.7-fold with damage. Unmodified peptides from the cytochrome C subunit CYC1 increased in abundance with UV damage, from 2.4- to 8.4-fold. An IkBa Ser32 phosphopeptide decreased 2.5-fold with UV treatment, while a triply phosphorylated IkBa peptide (Ser32, Ser36, Tyr42) decreased very slightly with UV (-1.4-fold). Other changes highlighted in Figure 5A were not consistent across all peptides, for example DNA-PK is highlighted in green, though only one unmodified peptide changed >2.5-fold, while 29 other unmodified peptides from DNA-PK did not. Similarly, only two out of 16 LDH-A peptides increased >2.5-fold with damage (see Table S4).

## 3. Discussion

### 3.1. Novel PTMScan Direct Reagents

Figures 1 and 2 highlight the capabilities of the new PTMScan Direct reagents to assess particular signaling pathways in detail. The DNA Damage/Cell Cycle Reagent provides an in-depth survey of proteins responsible for various functions in both normal cell cycle progression (DNA replication proteins, transcriptional regulators, cyclin dependent kinases and associated cyclins, *etc.*) as well as proteins that function in regulating the DNA damage response (checkpoint proteins, such as ATM, ATR, Chk1, Chk2 and ATRIP, stress responsive kinases, such as p38 MAPK and JNK, DNA Repair/Recombination factors, *etc.*). The Apoptosis/Autophagy Reagent allows the study of autophagolytic pathway markers, such as AKT, PI3K, PKC and ATG proteins, as well as apoptotic proteins, such as Fas and TNF receptors, caspases, BAD and Bcl proteins, cytochrome c and c-Jun. The diversity of proteins that can be monitored with these reagents make them powerful tools in the study of many signaling areas, including the response to UV damage of DNA. Further information about both reagents, including complete lists of proteins/sites validated, as well as sample data, can be found at http://www.cellsignal.com/services/direct_overview.html (Cell Signaling Technology, Danvers, MA, USA).

### 3.2. DNA Damage Response Signaling

The PTMScan Direct data in Figure 4 shows that the UV treatment of HeLa cells was sufficient to activate DNA damage checkpoint signaling, consistent with previous results [40]. The DNA Damage/Cell Cycle Reagent was designed to allow detection and quantification of both phosphorylated and unmodified peptides for the core DNA damage checkpoint proteins ATM, ATR, Chk1 and Chk2. This allowed differentiation between increased phosphorylation of a static level of each protein *versus* an increase in the total protein level, with no change in the fraction modified. The observation that there was no change in abundance of any of the unmodified peptides indicates that levels of these proteins remained consistent, while their phosphorylation states changed. Phosphorylation of ATM at Ser1981 increased with damage, as did phosphorylation at multiple sites on the effector kinases Chk 1 (Ser308, Ser317, Ser345) and Chk2 (S379).

Interestingly, the phosphopeptide containing Ser428 of ATR did not increase in abundance with damage, even though ATR is known to respond to UV damage and Ser428 phosphorylation has increased with UV treatment in other systems (www.cellsignal.com, Cell Signaling Technology, Danvers, MA, USA). The PTMScan Direct data, however, were consistent with the western blotting results in Figure 4E, where no change in signal was observed −/+ UV treatment using the ATR Ser428 antibody. This suggests the lack of response was not due to any technical limitations of PTMScan Direct, but rather a real reflection of the activation state of the samples. It should also be noted that, while Ser1981 of ATM is a critical site of phosphorylation and activation of ATM [82], Ser428 of ATR does not function in an analogous manner. There is another site on ATR, Thr1989, that is postulated to fulfill a similar role to Ser1981 of ATM [83–85]. Thus, no change in Ser428 phosphorylation may not indicate a lack of ATR activity in the study. The phosphorylation of Chk1 at ATR-dependent sites [86] also suggests ATR may be active, but not robustly phosphorylated, at Ser428 at the time point the cells were harvested.

There are other lines of evidence to indicate activation of the DNA damage checkpoint beyond ATM/ATR-Chk1/Chk2 signaling in this study. DNA-PK phosphorylation increased in response to UV damage at Ser3205. This is a site previously identified and responsive to DNA damage [87,88]. Activation of DNA-PK can affect DNA repair activities, as well as checkpoint signaling [89,90], and phosphorylation of this site may affect the activity of DNA-PK in these processes. Histone H2AX is a histone variant that is recruited to sites of DNA damage and phosphorylated, and it is thought to act as a scaffold for the binding of other checkpoint proteins at the DNA lesion [76]. H2AX phosphorylation increased with UV damage at Thr137 and Ser140, sites known to be phosphorylated by DNA-PK, JNK and p38 MAPK [91–95], all of which were phosphorylated and activated by UV treatment in this study. A decrease in p21cip1 peptides (both unmodified and phosphorylated at Ser130) was observed upon DNA damage. Changes to all p21cip1 peptides suggest regulation at the protein level in response to UV, rather than specific changes to a phosphorylation site. Previous studies have suggested a p53-dependent increase in p21cip1 levels in response to DNA damage [78,96], the opposite of what is observed here. Numerous reports, however, have found a dose- and time point-dependent UV-induced decrease in p21 protein levels in a variety of cellular systems [5,97–100], consistent with the results of this study. A p53-dependent increase in p21cip1 level may only occur at later time points post-damage than the two-hour harvest time employed here.

In addition to activation of canonical DNA damage checkpoint signaling, the stress responsive MAP kinases p38 and JNK were also activated by UV treatment. These kinases were identified phosphorylated at their activation loop sites (Thr180/Tyr182 for p38, Thr183/Tyr185 for JNK), which correlate with kinase activity and an increase in phosphorylation in response to UV damage [71,73,101]. Western blotting in Figure 4C also confirmed that the changes observed in PTMScan Direct were due to changes in phosphorylation state, rather than total protein level for both p38 MAPK and JNK.

### 3.3. Induction of Apoptosis

Figure 5 highlights the changes in peptide abundance observed using the Apoptosis/Autophagy Reagent. Most notable among these changes are the increased abundance of caspase peptides corresponding to cleaved, activated caspases. These increases were confirmed with the western blotting shown in Figure 5C, where the cleaved forms of caspases 3, 7 and 8 are more intense in the UV damaged sample than the control. It is also interesting to note that the phosphorylated forms of caspase 7 and caspase 9, but not caspase 3, increased with UV treatment. These phosphorylation events are not well characterized, but were previously identified in mitotic cells [102,103]. The biological impact of phosphorylation of caspases at residues close to their cleavage sites is not known, but may affect caspase activity, localization or protein-protein interactions.

Other peptides beyond those from caspases showed quantitative changes with UV treatment using the Apoptosis/Autophagy Reagent. Phosphopeptides from both c-Jun and JunD increased in abundance with UV damage. Ser63 and Ser73 of c-Jun and Ser100 of JunD are all JNK phosphorylation sites [72,73,104], and c-Jun is known to be induced by UV treatment [72,105,106]. As observed in Figure 4, JNK was activated by UV damage, providing a rationale for the increase in Jun phosphorylation. An IkBa peptide phosphorylated at Ser32 decreased with UV damage. Phosphorylation of IkBa increases upon UV damage [107–109], the opposite of what was observed in this study. UV damage-induced phosphorylation of IkBa, however, also leads to its ubiquitination and proteasomal degradation [110–112]. Thus, the decreased abundance of IkBa peptides in this study is likely due to a proteolysis-mediated decrease in IkBa protein levels.

Unmodified peptides from the cytochrome C subunit CYC1 also increased with UV treatment, though the reason for the increase is unknown. It is unlikely that it is due simply to release of CYC1 from mitochondria, as the lysis protocol used is known to free mitochondrial proteins (data not shown), and the related cytochrome C subunit CYCS does not show an increase in protein level with DNA damage. UV may therefore affect new transcription of CYC1 or interfere with its degradation, allowing accumulation in cells.

## 4. Experimental Section

### 4.1. Overview

PTMScan Direct is adapted from the PhosphoScan method developed at Cell Signaling Technology [47]. The method and reagent validation strategy are previously described [67].

### 4.2. Cell Lines

HCT-116, HeLa, NCI-H1703, NCI-H1299, Jurkat, T47D and A549 cells were from the American Type Culture Collection (Manassas, VA, USA). MKN-45 cells were from DSMZ (German Collection of Microorganisms and Cell Cultures). NCI-H3255 cells were from Dr. Lewis Cantley (Harvard Medical School, Boston, MA, USA). Etoposide treatment was for 6 h with 25 μM. Sodium pervanadate treatment was for 20 min with 50 ng/mL. Pervanadate treated cells were combined to create a cell line mix “CST Cell Line” in Tables S1–S4 to be run for validation of the reagents and as a control sample for all subsequent studies. Cells were cultured in recommended media supplemented with 10% fetal bovine serum and penicillin/streptomycin at 37 °C with 5% CO_2_. UV treatment was performed in a Stratagene 2400 Stratalinker at 50 mJ/cm^2^. Cells were treated with the media and plate cover removed. Then, the media was re-added, and cells were incubated for 2 h prior to harvesting. Peptides from 10 mg total soluble protein were used for a single immunoprecipitation, corresponding to roughly 1 × 10^8^ cells. Preparation of tryptic peptides from cellular lysates for immunoaffinity purification (IAP) was performed as previously described [67].

### 4.3. LC-MS/MS Analysis

Immunoprecipitated peptides were resuspended in 0.125% formic acid and separated on a reversed-phase C_18_ column (75 mm ID × 10 cm) packed into a PicoTip emitter (~8 mm ID) with Magic C_18_ AQ (100 Å × 5 mm). Peptides were eluted using a 72 min linear gradient of acetonitrile in 0.125% formic acid delivered at 280 nL/min. Tandem mass spectra were collected in a data-dependent manner with an LTQ-Orbitrap Velos or LTQ-Orbitrap Elite mass spectrometer running XCalibur 2.0.7 SP1 using a top-twenty MS/MS method, a dynamic repeat count of one and a repeat duration of 30 s. Real time recalibration of mass error was performed using lock mass with a singly charged polysiloxane ion *m*/*z* = 371.101237 [113]. MS/MS spectra were evaluated using SEQUEST 3G and the SORCERER 2 platform (v4.0, Sage-N Research, Milpitas, CA, USA) [70]. Human samples were searched against the NCBI homo sapiens FASTA database, updated on 27 June 2011. Results were filtered using a mass accuracy of +/− 50 ppm for precursor ions and 1 Da for product ions. Enzyme specificity was limited to trypsin, with at least one K or R terminus required per peptide and up to four mis-cleavages allowed. Cysteine carboxamidomethylation, specified as a static modification, oxidation of methionine residues, was allowed, and phosphorylation was allowed on serine, threonine and tyrosine residues. Reverse decoy databases were included for all searches to estimate false positive rates, and data was filtered using a 5% false discovery rate in the Peptide Prophet module of SORCERER 2. Search results were further filtered by mass accuracy based on clustering of forward database assignments (−/+ 5 ppm). Results were also filtered using criteria specific to each PTMScan Direct Reagent, including whether a particular peptide was targeted by an antibody in the reagent or homologous to a target, a minimum MS1 intensity filter of 20,000 in at least one sample run, and a higher abundance of the peptide in the PTMScan Direct Reagent IAP than in a control IAP using empty Protein G beads.

Label-free quantification was performed using proprietary software as previously described [41,42,67]. MS1 peak intensities across all samples were retrieved from the ion chromatogram files using a mass precision of −/+ 5 ppm and a retention time window of 5 min. Retention time warping (or chromatographic alignment) was performed across binary comparisons to allow retrieval of the correct peak intensity. Peak intensities for all peptides that changed in abundance between treatments were manually reviewed in the ion chromatogram files to ensure accuracy and where necessary peak height measurements were replaced with peak areas. The pervanadate treated cell line mixture (“CST Cell Line” in Supplementary Tables S1–S4) was run as a separate sample with each study as a rich source of modified peptides. This allowed the label-free quantification software to search for all validated peptides across the experimental samples for a more complete quantitative analysis.

### 4.4. Data Analysis

Protein-protein interactions from the STRING database (Version 9.0 [69]) were limited to the experimental, database and text-mining interaction categories with a minimum score of 0.700 (“high scoring” interactions in STRING). Interactions were imported into Cytoscape v2.8.1 for pathway map generation.

### 4.5. Western Blotting

Protein concentrations were determined by Bradford assay using Coomassie Plus Protein Assay Reagent (Pierce), and total protein was normalized between samples. Samples were mixed with SDS-PAGE sample buffer (#7723, Cell Signaling Technology, Danvers, MA, USA) and run on 4%–20% gradient tris-glycine gels (Invitrogen). Proteins were transferred to nitrocellulose (Millipore) and blocked for 1 h in 5% nonfat dry milk (Sigma) in TBS. All primary and secondary antibodies were from Cell Signaling Technology and were used according to standard protocols available at www.cellsignal.com. Blots were developed on the Odyssey near-infrared imaging system (LI-COR). Gels were stained with GelCode Blue Stain Reagent (Thermo Scientific, USA #24592) and destained with deionized H_2_O.

## 5. Conclusions

The PTMScan Direct method used to evaluate signaling in response to UV damage of DNA in HeLa cells is broadly applicable across biological systems. In this study, the reagents allowed detection of endpoints indicative of activation of DNA damage checkpoint signaling and onset of apoptosis, both of which were confirmed by western blotting. Future work could include varying the UV dose or harvest time or using different cell lines. This could provide both a dose response and a temporal view of DNA damage signaling and induction of apoptosis in response to UV treatment.

PTMScan Direct Reagents can also be used to study other signaling areas, such as Akt/PI3K signaling, Ser/Thr and Tyr Kinase activation, or look broadly at critical signaling proteins across many different pathways using the PTMScan Direct: Multipathway Reagent [67]. These reagents are, therefore. powerful tools for the study of diseases, such as cancer or neurodegeneration, normal cellular processes, such as growth, development and differentiation, and the study of novel inhibitors and therapeutic agents to determine inhibitory profiles and potential off-target effects. The method combines the specificity of antibody-based methods with the ability to assay a large number of endpoints typical of LC-MS-based proteomic analysis.

## Abbreviations

CST: Cell Signaling Technology
DDR: DNA damage response
IAP: Immunoaffinity purification
LC-MS/MS: Liquid chromatography tandem mass spectrometry
PI3K: Phosphatidylinositol 3-kinase
PTM: Post-translational modification

## Supplementary Files

Supplementary File 1 Supplementary Materials (ZIP, 5751 KB)
